# Prospective collection of bleeding rate and factor usage in participants with hemophilia A and B in a noninvestigational product study

**DOI:** 10.1016/j.rpth.2026.106870

**Published:** 2026-07-16

**Authors:** Kaan Kavakli, Laurent Frenzel, Ali Bulent Antmen, Hazzaa Alzahrani, Davide Matino, Andrew D. Leavitt, Margareth C. Ozelo, Jan Astermark, Young-Shil Park, Robert Klamroth, Adam Cuker, Barbara A. Konkle, John McKay, Delphine Agathon, Li-Jung Tseng, Maria de los Angeles Resa, Jasmine Healy, Lisa J. Wilcox, Frank Plonski

**Affiliations:** 1 Division of Hematology, Department of Pediatrics, Ege University Faculty of Medicine, Izmir, Turkey; 2 Department of Haematology, Institut Necker, Paris, France; 3 Acibadem Adana Hospital, Adana, Turkey; 4 Adult Haematology, King Faisal Specialist Hospital and Research Centre, Riyadh, Saudi Arabia; 5 Thrombosis and Atherosclerosis Research Institute, McMaster University, Hamilton, Ontario, Canada; 6 Department of Medicine, McMaster University, Hamilton, Ontario, Canada; 7 Departments of Medicine and Laboratory Medicine, University of California San Francisco, San Francisco, California, USA; 8 Hemocentro UNICAMP, Department of Internal Medicine, School of Medical Sciences, University of Campinas, Campinas, Brazil; 9 Department of Hematology, Oncology and Radiation Physics, Skåne University Hospital, Malmö, Sweden; 10 Department of Translational Medicine, Lund University, Malmö, Sweden; 11 Department of Pediatrics, Kyung Hee University Hospital at Gangdong, Kyung Hee University College of Medicine, Seoul, South Korea; 12 Vivantes Hospital, Friedrichshain, Berlin, Germany; 13 Institute of Experimental Hematology and Transfusion Medicine, University Hospital Bonn, Medical Faculty, University of Bonn, Germany; 14 Department of Medicine and Department of Pathology and Laboratory Medicine, Perelman School of Medicine, University of Pennsylvania, Philadelphia, Pennsylvania, USA; 15 Washington Center for Bleeding Disorders and the University of Washington, Seattle, Washington, USA; 16 Pfizer Inc, Pearl River, Connecticut, USA; 17 Pfizer Inc, Paris, France; 18 Pfizer Inc, New York, New York, USA; 19 Pfizer Canada ULC, Kirkland, Quebec, Canada; 20 Pfizer Inc, Collegeville, Pennsylvania, USA

**Keywords:** clinical study, data collection, hemophilia A, hemophilia B

## Abstract

**Background:**

We report efficacy and select safety outcomes of current factor prophylaxis of adults with moderately severe to severe hemophilia A (HA) or hemophilia B (HB) in a large, prospective, noninvestigational product study.

**Objectives:**

This study established prospective efficacy data on factor (F)VIII/FIX prophylaxis in the usual care setting.

**Methods:**

This prospective, open-label, noninvestigational product, multicenter study (BENEGENE-1) included HA and HB cohorts. Participants remained on their current factor replacement therapy. The primary end point was annualized bleeding rate. Secondary end points included annualized infusion rate of FVIII/FIX replacement therapy. Safety data were also collected.

**Results:**

In total, 241 individuals with HA and 333 with HB were screened, and 101 and 111, respectively, were enrolled in this study. The most common reason for screen failure was adeno-associated virus neutralizing antibody positivity, accounting for 82.1% and 92.3% of screen failures in the HA and HB cohorts, respectively. The corresponding overall mean (SD; range) follow-up duration was 310.8 (209.2; 14-948) and 498.6 (293.4; 117-1269) days. Mean (SD) total annualized bleeding rate and annualized infusion rate were 6.1 (10.6) and 127.1 (51.8) for participants with HA and 4.5 (9.4) and 62.8 (33.3) for participants with HB, respectively. No new safety concerns were identified with FVIII/FIX replacement therapy.

**Conclusion:**

This study provides a robust, prospective, informative dataset on bleeding rates in participants receiving factor replacement therapy over a mean follow-up of ∼1 year. Although FVIII/FIX prophylaxis was generally well tolerated, bleeding rate data illustrate the limitations of current standard-of-care factor prophylaxis.

## Essentials

• BENEGENE-1 looked at the efficacy and safety of factor prophylaxis in people with hemophilia.
• This large noninvestigational product study included 101 people with hemophilia A and 111 people with hemophilia B.
• Bleeding rates were comparable with those of other studies, with no new safety concerns identified.
• Results provide robust data for internal control use in subsequent phase 3 gene therapy trials.

## Graphical abstract

**Figure:**
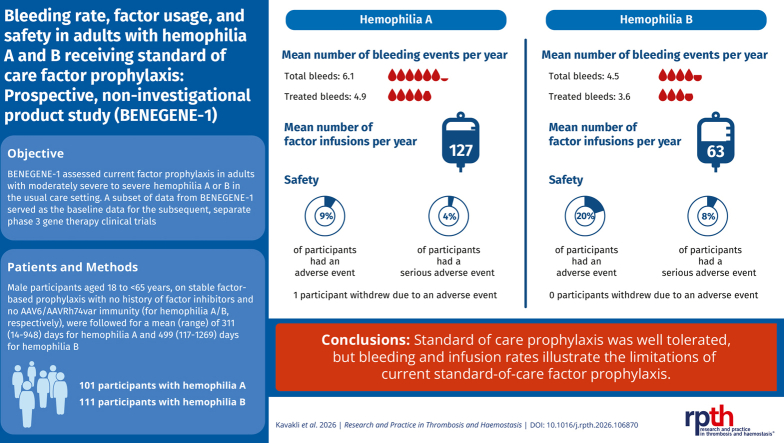

## 1 Introduction

Historically, standard of care for people with severe hemophilia included intravenous factor replacement therapy, either prophylactically at regular scheduled intervals to reach certain trough levels of factor activity or on-demand in the event of an acute bleed [1,2]. Prophylaxis frequency ranges from daily to once weekly for people with hemophilia A [3] and 2 to 3 times per week to twice per month for those with hemophilia B, depending on treatment half-life and disease severity [2]. Standard of care has evolved to include prophylaxis via subcutaneous injections of the nonfactor product emicizumab for hemophilia A [4] and marstacimab, concizumab, or fitusiran for both hemophilia A and B [[5], [6], [7]], with dosing frequencies ranging from daily to once every 2 months. Dose schedules for prophylactic regimens can be highly burdensome and affect quality of life and mental health, which further hinders adherence to prophylaxis [8,9].

Gene therapy is a novel treatment modality for people with hemophilia that works via gene transfer—typically using adeno-associated virus (AAV) vectors—to hepatocytes, where the clotting factor is then expressed and secreted into the circulation [10]. By providing continuous production of functional clotting factor, gene therapy may circumvent the need for recurrent factor replacement therapy. Two AAV5-based gene therapies are currently approved for persons with hemophilia—valoctocogene roxaparvovec [11] for those with hemophilia A and etranacogene dezaparvovec [12] for those with hemophilia B. Giroctocogene fitelparvovec and fidanacogene elaparvovec are AAV6 and AAV serotype rh74 (AAVRh74var)-based gene therapies for hemophilia A [13] and B [14], respectively. The phase 3 studies in participants with hemophilia A (AFFINE; NCT04370054) and hemophilia B (BENEGENE-2; NCT03861273) demonstrated significant reductions in the annualized bleeding rate (ABR) vs routine factor prophylaxis [15,16]. Both phase 3 studies required robust baseline data for bleeding outcomes, infusion rates, and safety with routine prophylactic factor replacement therapy.

The BENEGENE-1 (NCT03587116) study was a large, prospective, noninvestigational product study of the efficacy and select safety outcomes of current factor prophylaxis in the usual care setting of adults with moderately severe to severe hemophilia A or hemophilia B. A subset of data from BENEGENE-1 served as the baseline data for the subsequent, separate AFFINE [15] and BENEGENE-2 [16] interventional gene therapy dosing clinical trials. Thus far, only the outcomes for the subgroup of participants who subsequently received interventional gene therapy in AFFINE (*n* = 75) or BENEGENE-2 (*n* = 45) have been reported [15,16]. This is the first complete reporting of the full BENEGENE-1 study population that includes an additional 92 participants who enrolled in BENEGENE-1 but were not included in the subsequent AFFINE or BENEGENE-2 interventional gene therapy studies. Overall, data are presented for 101 participants with hemophilia A (compared with 75 participants who received giroctocogene fitelparvovec in AFFINE) [15] and 111 participants with hemophilia B (more than double the 45 participants who received fidanacogene elaparvovec in BENEGENE-2) [15,16]. Moreover, the analysis of data from this study provides an in-depth assessment of the unmet need and heterogeneity of outcomes in a group of individuals with hemophilia A or B receiving prophylactic factor replacement therapy but seeking a change from standard factor–based prophylaxis regimens.

## 2 Methods

### 2.1 Study design and participants

**Figure 1:**
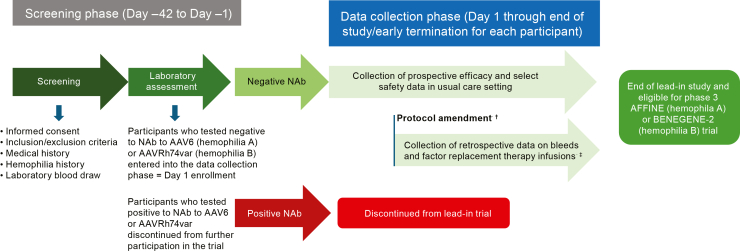
Study schema. ^†^Protocol amendment 5: Once the required number of participants treated with gene therapy was reached in the respective AFFINE and BENEGENE-2 interventional clinical trials, a protocol amendment was issued, allowing <6 months of follow-up prior to screening for phase 3 study enrollment. ^‡^Collection of retrospective data on numbers of bleeds and factor replacement therapy infusions within the 12 months prior to screening. AAV6, adeno-associated virus vector 6; AAVRh74var, AAV serotype rh74; NAb, neutralizing antibody.

This prospective, open-label, noninvestigational product, multicenter study (BENEGENE-1) included 2 cohorts: 1 for hemophilia A and 1 for hemophilia B. The study consisted of 2 periods (Figure 1): the screening period (day −42 to day −1) to assess eligibility, including AAV neutralizing antibody (NAb) status (relevant for subsequent eligibility and enrollment into the gene therapy dosing studies), and for eligible participants, the data collection period (day 1 through end of study or early termination for each participant). Participants remained on their current factor replacement therapy using their usual prophylaxis regimen. Recombinant standard half-life (rSHL), recombinant extended half-life (rEHL), or plasma-derived factor products were allowed. Bypassing or nonfactor agents were not allowed.

Participants were followed for a minimum of 6 months prior to being eligible for screening and potential enrollment into the subsequent phase 3 AFFINE and BENEGENE-2 gene therapy studies. Protocol amendment 5 was issued after the required number of participants treated with gene therapy was reached according to the statistical plan for primary efficacy analyses (*n* = 50 and *n* = 40 for AFFINE and BENEGENE-2, respectively). This amendment allowed <6 months of prospective follow-up prior to screening for phase 3 study enrollment.

Full eligibility criteria are in the Supplementary Material. Participants were men, aged 18 to <65 years with factor (F)VIII of ≤1% (hemophilia A) or FIX of ≤2% (hemophilia B), on a stable factor-based prophylaxis with no history of factor inhibitors. Key exclusion criteria were detectable anti-AAV6 and anti-AAVRh74var NAbs (for hemophilia A and B, respectively), unstable/clinically significant liver disease, and active hepatitis B or C viral infection. Participants with well-controlled HIV were eligible for inclusion.

The study was conducted in accordance with the International Conference on Harmonization Guideline for Good Clinical Practice and the Declaration of Helsinki. The protocol was approved by the institutional review board of the participating centers. Each patient provided signed informed consent prior to enrollment.

### 2.2 Study end points and assessments

#### 2.2.1 Efficacy end points

The primary end point was ABR. Data on treated and untreated bleeds were collected to obtain ABR_total_ (based on total number of treated and untreated bleeds) and ABR_treated_ (based on number of treated bleeds). Bleed definitions are provided in Supplementary Table 1. Secondary end points were annualized infusion rate (AIR) of exogenous FVIII/FIX replacement therapy, dose and total factor consumption, and the number of bleeding events (spontaneous and/or traumatic).

#### 2.2.2 Safety end points

Safety data comprised events meeting safety reporting requirements, including any research-related injury arising through blood draw collection during screening, nonserious adverse events (AEs) leading to study withdrawal/discontinuation or necessitating unscheduled clinic visits, and serious AEs (SAEs). Data for events of special interest (FVIII/FIX inhibitors, thrombotic events, and factor hypersensitivity events) were reported.

#### 2.2.3 Assessments

Bleeding events and factor usage were entered into an eDiary (Supplementary Material). In participants who were enrolled after protocol amendment 5, which permitted a shorter than 6-month follow-up period after the required number of treated participants in the respective treatment protocols were met, retrospective data on numbers of bleeds and factor replacement therapy infusions within 12 months prior to screening were also collected (hereafter termed the retrospective analysis set). Participants in the retrospective analysis set had both retrospectively collected (as part of the hemophilia history collected at screening) and prospectively collected bleeding and infusion data. As such, bleeding outcomes were assessed in this analysis set on both prospective (main analysis) and retrospective (exploratory) data.

### 2.3 Statistical analyses

The full analysis, safety, efficacy, and retrospective analysis sets are described in the Supplementary Appendix. Missing data were not imputed. All data were analyzed descriptively. A post hoc analysis evaluated ABR and AIR in participants who did not enroll or were not dosed in the subsequent phase 3 gene therapy studies.

## 3 Results

### 3.1 Hemophilia A cohort

#### 3.1.1 Study population

**Figure 2:**
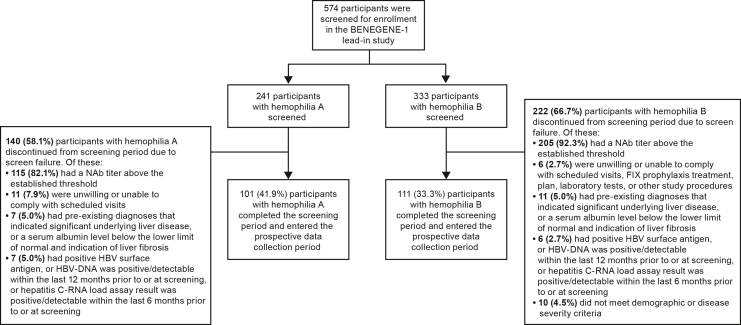
Study disposition. Note: Multiple reasons for discontinuation could be recorded so the total for screen failures in the hemophilia B cohort was >100%. HBV, hepatitis B virus; NAb, neutralizing antibody.

For the hemophilia A cohort, the study was conducted between September 17, 2019, and July 24, 2023, which included the period during the COVID-19 pandemic (Supplementary Material). Overall, 241 individuals with hemophilia A were screened at 35 centers across 18 countries/regions (Supplementary Table 2); 101 (41.9%) participants entered the prospective study and 140 (58.1%) discontinued due to screen failure. The most frequently reported reason for ineligibility was AAV6 immunity, accounting for 115 screen failures (47.7% of those screened; 82.1% of screen failures) (Figure 2). The AAV6 NAb positivity rate in the full analysis set was 48.1% (Table 1). The highest rate was in South America (70.6%), whereas the lowest rate was in Australia (0.0%; *n* = 2), followed by North America (14.3%). Rates for other regions ranged between 31.7% and 63.9%.

**Table 1:** Summary of AAV6 neutralizing antibody positive rate (hemophilia A cohort) and AAVRh74var NAb positive rate (hemophilia B cohort) by region—full analysis set^a^.

Region	AAV6 NAb positive rate (hemophilia A cohort)				AAVRh74var NAb positive rate (hemophilia B cohort)			
	*N*	*n*	Positive rate (%)	95% CI^b^ (%)	*N*	*n*	Positive rate (%)	95% CI^b^ (%)
Australia	2	0	0	0.0-84.2	20	10	50.0	27.2-72.8
Europe	60	19	31.7	20.3-45.0	84	46	54.8	43.5-65.7
South America	17	12	70.6	44.0-89.7	23	15	65.2	42.7-83.6
North America	21	3	14.3	3.0-36.3	24	12	50.0	29.1-70.9
Middle East	103	58	56.3	46.2-66.1	119	83	69.7	60.7-77.8
Asia	36	23	63.9	46.2-79.2	62	39	62.9	49.7-74.8
Total	239	115	48.1	41.6-54.7	332	205	61.7	56.3-67.0

AAV6, adeno-associated virus vector 6; AAVRh74var, AAV serotype rh74; CI, confidence interval; *N*, number of participants for each region, used for positive rate calculation in each region; *n*, number of NAb-positive participants; NAb, neutralizing antibody.

a Full analysis set: all consenting participants who had a blood sample collected and quantitatively assayed for AAV6/AAVRh74var immunity.

b CI was calculated by using the exact Clopper–Pearson method.

The mean (range) age was 31.8 (18-64) years (Table 2). Participants with hemophilia A were predominantly White (*n* = 78 [77.2%]) and not Hispanic or Latino (*n* = 76 [75.2%]). Target joints (defined as a major joint, eg, hip, elbow, wrist, shoulder, knee, and ankle) into which repeated bleeds occur (≥3 spontaneous bleeds into a single joint within a consecutive 6-month period) were identified in 47 (46.5%) participants. During the prospective period, rSHL FVIII was the most common class of factor replacement therapy, followed by rEHL FVIII (Supplementary Table 3). The most prescribed product was octocog alfa, followed by efmoroctocog alfa pegol and moroctocog alfa. The most common infusion regimens (based on regimen prescribed at baseline) were 2 times per week and 3 times per week (Supplementary Table 3).

**Table 2:** Baseline demographic and clinical characteristics—safety analysis set^a^.

Characteristic	Hemophilia A cohort (*n* = 101)	Hemophilia B cohort (*n* = 111)
Age (y)		
18-44	84 (83.2)	93 (83.8)
45-64	17 (16.8)	18 (16.2)
Mean (SD)	31.8 (11.2)	32.5 (11.3)
Median (range)	29.0 (18, 64)	30.0 (18, 61)
Geographic region		
Europe	34 (33.7)	34 (30.6)
North America	15 (14.9)	12 (10.8)
Middle East	34 (33.7)	31 (27.9)
Asia	12 (11.9)	18 (16.2)
South America	4 (4.0)	7 (6.3)
Australia	2 (2.0)	9 (8.1)
Race		
White	78 (77.2)	90 (81.1)^b^
Asian	17 (16.8)	18 (16.2)
Black or African American	6 (5.9)	2 (1.8)
Multiracial	0	1 (0.9)
Ethnicity		
Hispanic or Latino	4 (4.0)	4 (3.6)
Not Hispanic or Latino	76 (75.2)	91 (82.0)
Not reported	21 (20.8)	16 (14.4)
History of hepatitis		
Hepatitis B	5 (5.0)	14 (12.6)
Hepatitis C	24 (23.8)	28 (25.2)
HIV infection	2 (2.0)	3 (2.7)
Presence of target joints	47 (46.5)	56 (50.5)
No. of target joints		
0	54 (53.5)	55 (49.5)
1	22 (21.8)	32 (28.9)
2	13 (12.9)	10 (9.0)
≥3	12 (11.9)	14 (12.7)
Disease severity		
<1%	100 (99.0)	94 (84.7)
1%-2%	1 (1.0)^c^	17 (15.3)

Values are *n* (%) unless specified.

AAV6, adeno-associated virus vector 6; AAVRh74var, AAV serotype rh74; EHL-rFVIII, extended half-life recombinant FVIII; EHL-rFIX, extended half-life recombinant FIX; F, factor.

a Safety analysis set: All enrolled participants (negative for AAV6/AAVRh74var neutralizing antibodies) who entered the prospective data collection period.

b The race of 1 participant in the North America region was incorrectly reported as White; the correct race for this participant is Asian.

c Inclusion criteria for the hemophilia A cohort included documented FVIII activity ≤1% prior to baseline visit; 1 participant had FVIII activity level of 1%.

The mean (SD; range) follow-up duration was 310.8 (209.2; 14-948) days for the efficacy population (*n* = 96). Overall, 81 (84.4%) participants with hemophilia A had ≥6 months of follow-up (Supplementary Table 4), and 23 (24.0%) had ≥1 year of follow-up (Supplementary Table 4) because of a 1-year pause in recruitment for the subsequent phase 3 AFFINE study. Fifteen (15.6%) participants had <6 months of follow-up as they were recruited after implementation of protocol amendment 5 that permitted a shorter follow-up time; the mean (SD; range) follow-up duration for these participants was 83.8 (36.5; 14–152) days.

#### 3.1.2 Efficacy outcomes

**Figure 3:**
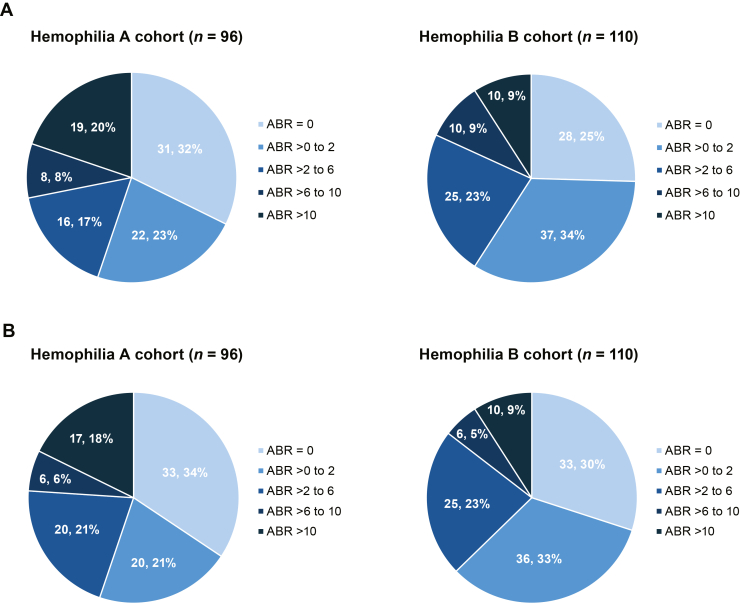
Categorical breakdown of annualized bleeding rates for (A) total and (B) treated bleeds during the prospective data collection period for the hemophilia A and hemophilia B cohorts—efficacy analysis set. All participants who were negative for AAV6/AAVRh74var neutralizing antibodies, and who participated in the prospective data collection period as part of their usual health care setting, were included in the efficacy analysis set. Each pie slice shows *n*, %. ABR, annualized bleeding rate.

Overall, 96 (95.0%) participants with hemophilia A were included in the efficacy analysis. Five (5.0%) participants were excluded from the efficacy analysis set due to protocol deviations that could have biased the interpretation of their efficacy data (eg, following an on-demand treatment instead of the prescribed prophylaxis treatment, a history of inhibitors, lack of compliance with documentation of bleeds, and/or prophylaxis replacement therapy administration). The mean (SD) ABR_total_ during the prospective period was 6.1 (10.6); values for ABR_treated_ were 4.9 (7.2) (Table 3). Overall, 31 (32%) participants had ABR_total_ = 0, and 19 (20%) had ABR_total_ > 10; 33 (34%) participants had ABR_treated_ = 0, and 17 (18%) had ABR_treated_ > 10 (Figure 3). The mean ABR_total_ was higher for spontaneous bleeds than for traumatic bleeds (Table 3). The mean (SD) AIR during the prospective period was 127.1 (51.8) with a mean (SD) annualized total FVIII replacement therapy consumption of 304,998 (153,932) IU (3903 IU/kg) (Table 4).

**Table 3:** Summary of annualized bleeding rate by bleeding event type and treatment status during the prospective data collection period—efficacy analysis set^a^.

Bleeding event type or treatment type	Annualized bleeding rate	
	Hemophilia A cohort (*n* = 96)	Hemophilia B cohort (*n* = 110)
Total bleeds (treated and untreated)		
Mean (SD)	6.1 (10.6)	4.5 (9.4)
Median (minimum-maximum)	1.8 (0.0-71.9)	1.7 (0.0-68.0)
All treated bleeds		
Mean (SD)	4.9 (7.2)	3.6 (7.6)
Median (minimum-maximum)	1.7 (0.0-42.8)	1.5 (0.0-66.5)
All untreated bleeds		
Mean (SD)	1.2 (6.5)	0.9 (4.0)
Median (minimum-maximum)	0.0 (0.0-60.9)	0.0 (0.0-40.1)
All spontaneous bleeds		
Mean (SD)	3.9 (7.6)	2.8 (6.4)
Median (minimum-maximum)	1.4 (0.0-40.0)	0.9 (0.0-53.9)
Treated spontaneous bleeds		
Mean (SD)	3.2 (6.0)	2.1 (3.8)
Median (minimum-maximum)	1.4 (0.0-40.0)	0.6 (0.00-24.0)
All traumatic bleeds		
Mean (SD)	2.3 (5.5)	1.7 (6.6)
Median (minimum-maximum)	0.0 (0.0-38.7)	0.00 (0.0-65.8)
Treated traumatic bleeds		
Mean (SD)	1.8 (3.9)	1.5 (6.4)
Median (minimum-maximum)	0.0 (0.0-24.8)	0.0 (0.0-64.3)

New treated bleeds and new untreated bleeds are used to calculate the annualized bleeding rate of treated bleeds and untreated bleeds, respectively, during the prospective data collection period. Concurrent new treated bleeds and concurrent new untreated bleeds of different sites on same date and time are counted only once under their corresponding treatment type.

AAV6, adeno-associated virus vector 6; AAVRh74var, AAV serotype rh74; *n*, number of participants in the specific analysis set.

a Efficacy analysis set: All participants who were negative for AAV6/AAVRh74var neutralizing antibodies and who participated in the prospective data collection period as part of their usual healthcare setting.

**Table 4:** Annualized infusion rate and factor consumption during the prospective data collection period—efficacy analysis set^a^.

	Hemophilia A cohort (*n* = 96)	Hemophilia B cohort (*n* = 110)
Annualized infusion rate	127.1 (51.8)	62.8 (33.3)
Annualized factor consumption (IU)	304,998 (153,932)	238,312 (127,817)
Annualized factor consumption per weight (IU/kg)	3903 (1850)	2911 (1395)

Note: Values are mean (SD). IU/kg was calculated using body weight (kg) at screening.

AAV6, adeno-associated virus vector 6; AAVRh74var, AAV serotype rh74; rFVIII, recombinant factor VIII product.

a Efficacy analysis set: All participants who were negative for AAV6/AAVRh74var neutralizing antibodies and who participated in the prospective data collection period as part of their usual health care setting.

Twenty-four participants with hemophilia A were enrolled in the retrospective analysis set (Supplementary Table 5). The mean ABRs for both retrospective and prospective data for this group of participants are shown in Supplementary Table 6.

#### 3.1.3 Safety

Of 101 participants with hemophilia A in the safety analysis set, 9 (8.9%) experienced ≥1 AE meeting safety reporting requirements, with a total of 10 AEs reported (Supplementary Table 7). SAEs were reported in 4 (4.0%) participants (Supplementary Table 8). One (1.0%) participant withdrew from the study owing to a moderate AE of depression after completing ≥6 months of prospective data collection. No AEs of special interest were reported.

### 3.2 Hemophilia B cohort

#### 3.2.1 Study population

For the hemophilia B cohort, the study was conducted between July 26, 2018, and December 13, 2024, which included the period during the COVID-19 pandemic (Supplementary Material). Overall, 333 participants with hemophilia B were screened at 65 centers across 18 countries/regions (Supplementary Table 2); 111 (33.3%) participants entered the prospective period, and 222 (66.7%) discontinued due to screen failure. The most frequently reported reason for ineligibility was AAVRh74var NAb positivity, accounting for 205 (92.3%) screen failures (Figure 2). NAb positivity rate in the full analysis set was 61.7% (Table 1). The highest rate was in the Middle East (69.7%) and lowest rates were in North America and Australia (50.0%).

The mean (range) age of participants with hemophilia B was 32.5 (18-61) years (Table 2). Participants were predominantly White (*n* = 90 [81.1%]) and not Hispanic or Latino (*n* = 91 [82.0%]). Target joints were identified in 56 (50.5%) participants. During the prospective period, rEHL FIX was the most common class of factor replacement therapy, followed by rSHL FIX (Supplementary Table 9). The most prescribed product was eftrenonacog alfa, followed by nonacog alfa. The most common infusion regimen (based on regimen prescribed at baseline) was once per week (Supplementary Table 9).

The mean (SD; range) follow-up duration was 498.6 (293.4; 117-1269) days in the efficacy population with hemophilia B (*n* = 110). Overall, 107 (97.3%) participants had ≥6 months of follow-up, with a mean (SD; range) follow-up duration of 508.7 (291.0; 184-1269) days; follow-up was ≥1 year in 63 of these participants and ≥2 years in 23 participants (Supplementary Table 4). For the 3 (2.7%) participants with <6 months of follow-up, due to recruitment after implementation of protocol amendment 5, mean (SD; range) duration was 136.3 (25.3; 117-165) days.

#### 3.2.2 Efficacy outcomes

Overall, 110 (99.1%) participants with hemophilia B were included in the efficacy analysis. One (0.9%) participant was excluded from the efficacy analysis set due to a protocol deviation that could have biased the interpretation of their efficacy data (lack of compliance with documentation of bleeds and/or prophylaxis replacement therapy administration). The mean (SD) ABR_total_ during the prospective period was 4.5 (9.5); values for ABR_treated_ were 3.6 (7.6) (Table 3). Twenty-eight (25%) participants had ABR_total_ = 0, and 10 (9%) had ABR_total_ > 10; 33 (30%) participants had ABR_treated_ = 0, and 10 (9%) had ABR_treated_ > 10 (Figure 3). The mean ABR_total_ was higher for spontaneous bleeds than for traumatic bleeds (Table 3). The mean (SD) AIR during the prospective period was 62.8 (33.3), with mean (SD) annualized total FIX replacement therapy consumption of 238,312 (127,817) IU (2911 IU/kg) (Table 4).

Thirty-nine participants enrolled in the retrospective analysis set (Supplementary Table 5). For this group of participants, the mean ABRs for both retrospective and prospective data are shown in Supplementary Table 6.

#### 3.2.3 Safety

Of 111 participants with hemophilia B in the safety analysis set, 22 (19.8%) experienced ≥1 AE, with a total of 44 AEs reported (Supplementary Table 10). SAEs were reported in 9 (8.1%) participants (Supplementary Table 11). No participants withdrew from the study owing to AEs. Overall, 2 (1.8%) participants reported 3 events of special interest in the category of factor hypersensitivity: none of these events were related to FIX replacement therapy. Of these events, 2 were of allergic dermatitis reported in 1 participant (1 event categorized as mild and the other as moderate), and the third event was pneumonitis (categorized as severe).

## 4 Discussion

This prospective, noninvestigational product study provides informative data on bleeding rates in participants receiving factor replacement therapy with a mean follow-up duration of ∼1 year across the hemophilia A and hemophilia B cohorts. This follow-up exceeds the planned 6-month duration and consequently provides 1 of the largest prospective follow-up studies for people with hemophilia, particularly for those with hemophilia B and those seeking gene therapy. As such, the study fulfilled its primary objective to establish prospective efficacy data on standard-of-care FVIII/FIX prophylaxis replacement therapy to provide robust data for internal control use in phase 3 AFFINE and BENEGENE-2 gene therapy studies.

A large proportion of participants in this study were not eligible for gene therapy (58.1% and 66.7% in the hemophilia A and B cohorts, respectively), predominantly owing to AAV6 or AAVRh74var immunity and, to a lesser degree, underlying liver disease, or active hepatitis B/C infection. The overall rate of AAV6 NAb positivity (48.1%) was consistent with the 48.7% seroprevalence rate reported in a large, global study assessing preexisting immunity against multiple AAV serotypes across 9 countries [17] and a rate of 46.2% in the overall adult population in another large, global AAV seroprevalence study across 10 countries [18]. AAVRh74var NAb positivity (61.7%) was higher than AAV6 and was consistent with recently reported global AAVRh74var seroprevalence rates (58.4%) [18]. These data not only demonstrate the representativeness of the study populations with respect to AAV immunity but also highlight the inherent challenges imposed by humoral immunity when assessing eligibility for gene therapy and the importance of continued development of novel vectors.

In the hemophilia A cohort, the mean ABR_treated_ was consistent with rates described in multiple interventional and observational studies [19]. Although fewer historical data are available for the ABR_total_, the rate observed in this study was similar to a recent global, prospective noninterventional study of persons with severe hemophilia A receiving prophylaxis (mean [SD], 4.81 [6.83]) [20]. A similar 20% rate difference for total vs treated bleeds was observed in interventional prophylaxis studies [21]. In the hemophilia B cohort, the mean ABR_total_ and ABR_treated_ (rate difference, 20%) were generally consistent with rates described in other studies evaluating FIX products [[22], [23], [24]]. The ABR_treated_ reported in this study for the hemophilia A and B cohorts in the retrospective period after protocol amendment 5 (implemented shortly after the enrollment pause due to COVID-19) were higher than many historical reports but similar to the mean ABR_treated_ reported in recent phase 3 trials for nonfactor products [[25], [26], [27]]. Overall, consistent data were observed between the retrospective and prospective periods for the retrospective analysis set, suggesting acceptable compliance in eDiary entries. However, the high ABRs observed in persons with hemophilia A and hemophilia B on routine prophylaxis highlight the importance of more effective prophylactic strategies to address bleeding and the underlying disease and treatment burden despite perceptions from real-world observations that bleed control has been optimized [28].

The ratio of untreated to treated bleeds in each cohort was similar (0.25 and 0.23 for hemophilia A and B, respectively). This indicates the majority of bleeds were treated with factor replacement therapy, a finding consistent with the lead-in factor prophylaxis periods for other hemophilia gene therapies [20,24]. In contrast, rates for some nonfactor therapies such as emicizumab have reported a higher proportion of untreated bleeding events across studies [29].

In this study, the mean ABRs for both total and treated bleeds were numerically higher in the hemophilia A (vs hemophilia B) cohort, but median ABRs were similar. Interestingly, a higher proportion of participants with hemophilia A had an ABR_total_ = 0 vs the hemophilia B cohort (32% vs 25%); however, the hemophilia A cohort also had more participants with high bleed rates (ie, ABR_total_ > 10) vs the hemophilia B cohort (20% vs 9%). This indicates substantial heterogeneity in bleeding phenotype and may also be influenced by heterogeneity in the underlying prophylaxis regimens.

Heterogeneity in disease burden (ABR) and treatment burden (AIR), coupled with a large proportion of participants with well-controlled disease (ABR = 0) suggests dissatisfaction with current factor prophylaxis, and desire for a new therapeutic option such as gene therapy likely involves multiple, complex motivational factors that cannot be explained simply by poor bleed control or perceived treatment burden. This is evident in the hemophilia B cohort where many participants with ABR = 0 were on a weekly to less frequent prophylactic regimen. Despite excellent bleed control and what might be considered a low-burden regimen, these participants were still actively seeking gene therapy. The desire for gene therapy in these cases may be motivated by a combination of the perceived burden of treatment and of the disease itself. There is the potential to achieve normalization of hemostasis through gene therapy and subsequent relief from treatment and disease burden [30]. The potential to live without the frustrations, psychological distress, and coping strategies involved with hemophilia treatment, even when it is effective, may be a key factor [31]. These data support continued innovation and development of new therapies, and further work is needed to better understand patient motivation for alternative therapies, such as gene therapy, that may provide a hemophilia-free mindset [31]. With time, those who have received gene therapy will be able to confirm whether they have achieved this goal.

No new safety concerns were identified with FVIII or FIX replacement therapy. Although the safety data are limited, they are consistent with published literature supporting the tolerability of factor replacement therapy [32].

There are several limitations of this study, particularly related to the potential for selection bias. The study potentially favored recruitment of those with higher ABRs and dissatisfaction with their current treatment given the future possibility of enrollment in the subsequent phase 3 gene therapy studies. Bleeds were self-reported by patients without imaging or independent verification. Additionally, the study only permitted prophylaxis with factor replacement therapies and did not include participants using nonfactor or on-demand factor therapies.

## 5 Conclusion

This large, prospective, noninvestigational product study provides robust informative data on bleeding rates in persons with hemophilia A or B receiving factor replacement therapy for a mean follow-up of ∼1 year. Although FVIII/FIX prophylaxis was well tolerated, with no emerging safety signals, bleeding rates illustrate the limitations and patient-to-patient variability of current standard-of-care factor prophylaxis. This study also suggests that the motivation to receive gene therapy is likely complex and multifactorial and that physicians should explore factors beyond bleed control and treatment burden to understand remaining unmet need for patients.
