# Supplementary material for: Prospective collection of bleeding rate and factor usage in participants with hemophilia A and B in a noninvestigational product study
Source: Res Pract Thromb Haemost. 2026 Jul 16;10(5):106870. doi: 10.1016/j.rpth.2026.106870 (PMC13474634; doi:10.1016/j.rpth.2026.106870)
Supplement: Supplementary file 1 [file mmc1.pdf]

SUPPLEMENTAL APPENDIX

Eligibility Criteria

Inclusion criteria

Hemophilia A population	Hemophilia B population
Males ≥18 and <65 years of age with moderately severe to severe hemophilia A and documented FVIII activity (≤1%) prior to baseline visit	Males ≥18 and <65 years of age with moderately severe to severe hemophilia B and documented FIX activity (≤2%) prior to baseline visit.
Previous experience with FVIII therapy (≥150 documented exposure days to a FVIII protein product such as recombinant, plasma-derived or extended half-life FVIII product)	Previous experience with FIX therapy (≥50 documented exposure days to a FIX protein product such as recombinant, plasma-derived or extended half-life FIX product)
Participants on FVIII prophylaxis replacement therapy (recombinant, plasma-derived or extended half-life FVIII product) must have the intention to remain on FVIII prophylaxis replacement therapy for the duration of the study	Participants on FIX prophylaxis replacement therapy (recombinant, plasma-derived or extended half-life FIX product) must have the intention to remain on FIX prophylaxis replacement therapy for the duration of the study
No known hypersensitivity to FVIII replacement product	No known hypersensitivity to FIX replacement product
No history of FVIII inhibitor (clinical or laboratory-based assessment) defined as a titer ≥0.6 BU/mL, regardless of the laboratory normal range, or any measured Bethesda inhibitor titer greater than the upper limit of normal for the laboratory performing the assay; clinically, no signs or symptoms of decreased response to FVIII administration	No history of FIX inhibitor (clinical or laboratory-based assessment) defined as a titer ≥0.6 BU/mL, regardless of the laboratory normal range, or any measured Bethesda inhibitor titer greater than the upper limit of normal for the laboratory performing the assay; clinically, no signs or symptoms of decreased response to FIX administration
Evidence of a signed and dated informed consent document indicating the participant has been informed of all pertinent aspects of the study	

Willing and able to comply with scheduled visits, factor prophylaxis treatment plan, laboratory tests and other study procedures
FIX, factor IX; FVIII, factor VIII.

Exclusion criteria

Participants with any of the following characteristics/conditions could not be included in the study.

1. Anti-AAVRh74var (previously referred to as AAV-Spark100) neutralizing antibody titer above the established threshold (at the cut-off titer [1:1] in serum samples) performed by a central laboratory during screening in hemophilia B participants or anti-AAV6 neutralizing antibody titer above the established threshold (4 [1:4 dilution] in serum samples) performed by a central laboratory during screening in hemophilia A participants.
2. Lack of participant compliance with documentation of bleeds and/or prophylaxis replacement therapy administration.
3. If there was no documentation regarding hepatitis status, as defined below, within the last 12 months prior to screening for hepatitis B and 6 months prior to screening for hepatitis C, then participants were required to have the following hepatitis testing performed at screening:
    a. **Hepatitis B screening (acute and chronic):**
    - HBsAg (also referred to as hepatitis B surface antigen), HBV-DNA viral assay (also referred to as a nucleic acid test for hepatitis B virus DNA), and Anti-HBc (also referred to as total hepatitis B core antibody).
    - A participant was not eligible if either HbsAg was positive or HBV-DNA was positive/detectable.
    - Anti-HBc was obtained in all participants for determination of whether the participant had prior hepatitis B. If the anti-HBc was positive and both HBsAg and HBV DNA were negative, this would be consistent with a *prior* infection and the participant was eligible for the study. Anti-HBc was obtained in all participants to discriminate between those with no prior hepatitis B and those with prior infection in the event of reactivation.

- One documented negative HBV-DNA viral load was sufficient to assess eligibility. A participant who was currently undergoing antiviral therapy for hepatitis B at the time of screening was not eligible.

b. Hepatitis C (acute or chronic):

- A participant who was currently undergoing antiviral therapy for chronic hepatitis C at the time of screening was not eligible.
- Participants treated with antiviral therapy for chronic hepatitis C must have completed antiviral therapy ≥6 months prior to screening and have had a negative HCV-RNA ≥6 months prior to screening.
- All participants (who were not currently undergoing antiviral therapy for chronic hepatitis C at the time of screening) must have had a single HCV-RNA load assay (also referred to as a nucleic acid test [NAT] for HCV-RNA) obtained during the 6 months preceding screening. This included participants with prior known chronic hepatitis C who have completed treatment with anti-viral therapy.
- A participant was not eligible if the HCV-RNA load assay result was positive/detectable.

4. Currently on antiviral therapy for hepatitis B or C at the time of screening.
5. Significant liver disease, as defined by pre-existing diagnosis of portal hypertension, splenomegaly, or hepatic encephalopathy.
    - All participants who did not have the listed preexisting diagnoses above must have had the following assessments performed within the last 12 months prior to screening or were tested for liver fibrosis status at screening: a serum albumin level below normal limits and/or significant liver fibrosis by any of the following diagnostic modalities: FibroScan score >8 kPa units OR FibroTest/FibroSURE >0.48.
6. Documented serological evidence of HIV 1 or HIV 2 with cluster of differentiation 4 positive (CD4+) cell count ≤200/mm^3 within the last 12 months prior to screening. Participants who were HIV-positive and stable, with an adequate CD4 count (>200/mm^3) and undetectable viral load (<50 gc/mL) documented within the preceding 12 months, and were on an antiretroviral drug regimen, were eligible to enroll. Participants who had not been tested within the prior 12 months of screening were tested for HIV status at screening.
7. History of chronic infection or other chronic disease that the investigator deemed an unacceptable risk. In addition, any participant with conditions

associated with increased thromboembolic risk such as known inherited or acquired thrombophilia, or a history of thrombotic events including but not limited to stroke, myocardial infarction, and/or venous thromboembolism, were excluded.

8. Any concurrent clinically significant major disease or condition the investigator deems unsuitable for participation or other acute or chronic medical or psychiatric condition including recent (within the past year) or active suicidal ideation or behavior or laboratory abnormality that may increase the risk associated with study participation or may interfere with the interpretation of study results and, in the judgment of the investigator, would have made the participant inappropriate for entry into this study. In addition, any participant with a history of a neoplasm (including hepatic malignancy) that required treatment (eg, chemotherapy, radiotherapy, immunotherapy) is excluded, except for adequately treated basal or squamous cell carcinoma of the skin or a surgically removed benign neoplasm not requiring chemotherapy, radiotherapy and/or immunotherapy. Any other neoplasm that has been cured by resection was discussed between the investigator and sponsor.
9. Participation in other studies if involving administration of investigational product(s) within the last 3 months prior to study entry and/or during study participation or in a previous gene therapy clinical study within the last 12 months prior to screening.
    - Participants already enrolled in this lead-in study were allowed to participate in the screening and baseline periods of either AFFINE or BENEGENE-2 protocols prior to their completion of the end of study visit in this lead-in study.
10. Any participant who previously received fidanacogene elaparvovec (hemophilia B) or giroctocogene fitelparvovec (hemophilia A) or any AAV gene-based therapy.
11. Participants using restricted therapies, such as bypassing agents and nonfactor treatment, antifibrinolytics, blood products such as red blood cells, platelets and fresh frozen plasma, medications known to inhibit platelet function, and concomitant use of another investigational product.
12. Any participant with a planned surgical procedure requiring FIX (hemophilia B) or FVIII (hemophilia A) surgical prophylactic factor treatment within 24 months of screening.

13. Investigator site staff members directly involved in the conduct of the study and their family members, site staff members otherwise supervised by the investigator, or participants who are Pfizer employees, including their family members, directly involved in the conduct of the study.

Assessments

Eligible participants were provided with a handheld eDiary device at the Day 1 visit to record their bleeding events and FVIII/FIX replacement therapy infusions until their end-of-study or early termination visit. Participants entered information on factor replacement therapy infusions, including date, time, reason for administration (on demand, preventative or prophylactic), name or category of the product used to treat the bleed, and dose (international units [IU]) and bleeding events (including treatment used [where applicable], date, time, type of bleeds [ie, trauma-related or spontaneous] ,and locations based on a body map) into the eDiary on an ongoing basis.

Analysis sets

Full analysis set	All consenting participants who had a blood sample collected and quantitatively assayed for AAV6/AAVRh74var immunity
Safety analysis set	All enrolled participants (negative for AAV6/AAVRh74var NAbs) who entered the prospective data collection period
Efficacy analysis set	All participants who were negative for AAV6/AAVRh74var NAbs, and who participated in the prospective data collection period as part of their usual health care setting
Retrospective analysis set	All participants who were enrolled under protocol amendment 5 and afterwards, fulfilled the inclusion/exclusion criteria and who had retrospective and prospective data collected

NAb, neutralizing antibody.

Impact of COVID-19

Hemophilia A cohort: There were no AEs related to COVID-19 that met the study safety reporting requirements, no disruptions or discontinuations due to COVID-19 occurred, and COVID-related protocol deviations were minimal.

Hemophilia B cohort: One mild AE of COVID-19 was reported (AE associated with an unscheduled clinic visit), no participants had their participation disrupted or discontinued due to COVID-19, and COVID-related protocol deviations were minimal.

Supplemental Table S1. Bleed definitions.

Bleed type	Definition
Treated bleed	An event necessitating administration of coagulation factor within 72 hours of signs or symptoms of bleeding; if a bleed during the prospective data collection period had treatment type "FVIII TREATMENT" (hemophilia A) or "FIX TREATMENT" (hemophilia B) and was treated with coagulation factor infusion within 72 hours, regardless of the reason of treatment (preventive, prophylaxis or rescue medication), it was considered to be a treated bleed
Untreated bleed	A bleeding event not necessitating administration of coagulation factor within 72 hours of signs or symptoms of bleeding
New treated bleed during the prospective data collection period	A bleed occurring >72 hours after stopping treatment from the original bleed for which treatment was initiated or a bleed occurring at a different site from the original bleed regardless of the time from last injection
New untreated bleed during the prospective data collection period	A bleed occurring >72 hours after the previous untreated bleed at the same site or occurring >72 hours after stopping treatment from the original bleed for which treatment was initiated, or an untreated bleed occurring at a different site from the original bleed regardless of the time

FIX, factor IX

Supplemental Table S2. Screened participants by region and study site.

	Hemophilia A cohort	Hemophilia B cohort
Australia	2	20
Europe	61	84
South America	17	23
North America	22	24
Middle East	103	119
Asia	36	63
All countries combined	241†	333‡

† In total, 241 patients with hemophilia A were screened at 35 centers across 18 countries/regions, including the United States and Turkey (5 sites each); Italy and Taiwan (3 site each); Australia, Brazil, Japan, South Korea and Spain (2 sites each); and Belgium, Canada, France, Germany, Greece, Israel, Saudi Arabia, Sweden and the United Kingdom (1 site each).

‡ In total, 333 participants with hemophilia B were screened at 65 centers across 18 countries/regions, including the United States (7 sites); Japan and Turkey (6 sites each); Australia, Brazil, Spain and Taiwan (5 sites each); France and Germany (4 sites each); Italy, South Korea and the United Kingdom (3 sites each); Canada, Greece and Saudi Arabia (2 sites each); and Belgium, Israel and Sweden (1 site each).

Supplemental Table S3. FVIII replacement therapy and regimen frequency during the prospective data collection period for the hemophilia A cohort (based on initial regimen prescribed at baseline) (*N* = 101)—safety analysis set.

**Product type**	***n* (%)**
Extended half-life rFVIII	31 (30.7)
Damoctocog alfa pegol	6 (5.9)
Efmoroctocog alfa pegol	18 (17.8)
Rurioctocog alfa pegol	5 (5.0)
Turoctocog alfa pegol	1 (1.0)
Unspecified	1 (1.0)
Standard half-life rFVIII	60 (59.4)
Beroctocog alfa	1 (1.0)
Lonoctocog alfa	1 (1.0)
Moroctocog alfa	14 (13.9)
Octocog alfa	31 (30.7)
Rurioctocog alfa	1 (1.0)
Simoctocog alfa	2 (2.0)
Turoctocog alfa	7 (6.9)
Unspecified recombinant	3 (3.0)
Plasma-derived FVIII	8 (7.9)
Emoclot	2 (2.0)
Haemoctin/Haemoctin SDH	2 (2.0)
Hemofil-M	1 (1.0)
Koate	2 (2.0)
Unspecified plasma-derived	1 (1.0)
Other, unspecified	2 (2.0)
Prophylactic regimen frequency	
≤1x weekly	8 (7.9)
2x weekly	35 (34.7)
3x weekly	39 (38.6)
Every other day	8 (7.9)
Daily	3 (3.0)
Other	7 (6.9)
Unspecified or Intermittent	1 (1.0)

FVIII, factor VIII; rFVIII, recombinant factor VIII product.

Supplemental Table S4. Duration of follow up—efficacy analysis set.

Duration of follow-up, months	Hemophilia A (*n* = 96)<br>*n* (%)	Hemophilia B (*n* = 110)<br>*n* (%)
<6 months	15 (15.6)	3 (2.7)
≥6 to <12 months	58 (60.4)	44 (40.0)
≥12 to <24 months	13 (13.5)	40 (36.4)
≥24 months	10 (10.4)	23 (20.9)

Supplemental Table S5. Baseline demographic and clinical characteristics—retrospective analysis set.[†]

Characteristic	Hemophilia A cohort (*n* = 24)	Hemophilia B cohort (*n* = 39)
Age, years		
18–44, *n* (%)	22 (91.7)	31 (79.5)
45–64, *n* (%)	2 (8.3)	8 (20.5)
Mean (SD)	25.5 (8.3)	34.3 (11.8)
Median (range)	22.5 (18, 50)	35.0 (18, 60)
Geographic region, *n* (%)		
Europe	1 (4.2)	14 (35.9)
North America	2 (8.3)	0
Middle East	17 (70.8)	5 (12.8)
Asia	1 (4.2)	11 (28.2)
South America	3 (12.5)	3 (7.7)
Australia	0	6 (15.4)
Race, *n* (%)		
White	20 (83.3)	26 (66.7)
Asian	2 (8.3)	11 (28.2)
Black or African American	2 (8.3)	1 (2.6)
Multiracial	0	1 (2.6)
Ethnicity, *n* (%)		
Hispanic or Latino	0 (0.0)	4 (10.3)
Not Hispanic or Latino	21 (87.5)	31 (79.5)
Not reported	3 (12.5)	4 (10.3)
Presence of target joints, *n* (%)	12 (50.0)	23 (59.0)

SD, standard deviation.

[†] Participants enrolled under Protocol Amendment 5 or later had collection of retrospective data (numbers of bleeds and infusions within the last 12 months prior to screening), which were not collected for participants enrolled under earlier versions of the protocol. These participants were also permitted to have a follow-up duration (prospective) of less than 6 months.

Supplemental Table S6. Summary of bleeding and infusion outcomes and factor replacement therapy consumption for the hemophilia A and hemophilia B retrospective analysis sets.

	Hemophilia A cohort			Hemophilia B cohort		
**Mean (SD)**	**Retrospective data collection period (*n* = 24)**	**Prospective data collection period (*n* = 24)**	**Combined collection periods (*n* = 24)**	**Retrospective data collection period (*n* = 39)**	**Prospective data collection period (*n* = 39)**	**Combined collection periods (*n* = 39)**
Annualized bleeding rate						
All bleeds	14.3 (18.2)	10.9 (16.7)	12.8 (16.3)	6.79 (10.4)	4.8 (10.9)	5.5 (8.3)
Spontaneous	-	7.0 (11.5)	-	-	2.8 (6.5)	-
Traumatic	-	4.1 (8.7)	-	-	1.7 (6.7)	-
Treated bleeds	9.8 (13.7)	7.6 (9.9)	9.0 (11.9)	5.42 (8.2)	4.2 (10.6)	4.7 (7.6)
Spontaneous	-	5.2 (9.1)	-	-	2.2 (3.9)	-
Traumatic	-	2.6 (4.6)	-	-	1.5 (6.5)	-
Annualized infusion rate	141.9 (50.5)	133.2 (56.7)	139.9 (52.5)	66.97 (30.5)	57.0 (21.0)	61.0 (23.1)
Factor therapy consumption	-	235,097 (67,977)	-	-	223,408 (85,995)	-

Note: (-) is not reported.
SD, standard deviation.

Supplemental Table S7. Incidence and severity of adverse events: hemophilia A cohort (*N* = 101)—safety analysis set.

	***n* (%)**			
**MedDRA Preferred Term**	**Mild**	**Moderate**	**Severe**	**Total**
Any AE	2 (2.0)	4 (4.0)	3 (3.0)	9 (8.9)
B-cell lymphoma	0	0	1 (1.0)	1 (1.0)
Cubital tunnel syndrome	0	1 (1.0)	0	1 (1.0)
Depression	0	1 (1.0)	0	1 (1.0)
Hand fracture	1 (1.0)	0	0	1 (1.0)
Hemarthrosis	0	1 (1.0)	0	1 (1.0)
Hemorrhoidal hemorrhage	0	0	1 (1.0)	1 (1.0)
Myalgia	0	1 (1.0)	0	1 (1.0)
Oropharyngeal pain	1 (1.0)	0	0	1 (1.0)
Upper gastrointestinal hemorrhage	0	1 (1.0)	0	1 (1.0)
Wound infection	0	0	1 (1.0)	1 (1.0)

Note: If a participant had more than one occurrence in the same preferred term event category, only the most severe occurrence was counted. Participants were counted only once per System Organ Class and Preferred Term. For the AE severity imputation algorithm, any missing severities were imputed as severe unless the participant experienced another occurrence of the same event for which severity was recorded. In this case, the reported severity is summarized. Includes all data collected from the time the participant provides informed consent through the duration of the study until their final study or early termination visit. MedDRA v26.0 coding dictionary was applied.

AE, adverse event; MedDRA, Medical Dictionary for Regulatory Activities.

Supplemental Table S8. Incidence and severity of serious adverse events: hemophilia A cohort (*N* = 101)—safety analysis set.

	***n* (%)**			
**MedDRA Preferred Term**	**Mild**	**Moderate**	**Severe**	**Total**
Any SAE	0	1 (1.0)	3 (3.0)	4 (4.0)
B-cell lymphoma	0	0	1 (1.0)	1 (1.0)
Hemorrhoidal hemorrhage	0	0	1 (1.0)	1 (1.0)
Upper gastrointestinal hemorrhage	0	1 (1.0)	0	1 (1.0)
Wound infection	0	0	1 (1.0)	1 (1.0)

Note: If a participant had more than one occurrence in the same preferred term event category, only the most severe occurrence was counted. Participants were counted only once per System Organ Class and Preferred Term. For the AE severity imputation algorithm, any missing severities were imputed as severe unless the participant experienced another occurrence of the same event for which severity was recorded. In this case, the reported severity is summarized. Includes all data collected from the time the participant provides informed consent through the duration of the study until their final study or early termination visit. MedDRA v26.0 coding dictionary was applied.

AE, adverse event; MedDRA, Medical Dictionary for Regulatory Activities; SAE, serious adverse event.

Supplemental Table S9. FIX replacement therapy and regimen frequency during the prospective data collection period for the hemophilia B cohort (based on initial regimen prescribed at baseline) (*N* = 111)—safety analysis set.

**Product type**	***n* (%)**
Extended half-life rFIX	65 (58.6)
Albutrepenonocog alfa	15 (13.5)
Eftrenonacog alfa	44 (39.6)
Nonacog beta pegol	6 (5.4)
Standard half-life rFIX	33 (29.7)
Nonacog alfa	32 (28.8)
Nonacog gamma	1 (0.9)
Plasma-derived FIX	10 (9.0)
AIMAFIX	2 (1.8)
Immunine	1 (0.9)
Octanine/Octanine F	6 (5.4)
Plasma-derived, unspecified	1 (0.9)
Unspecified FIX	3 (2.7)
Prophylactic regimen frequency	
<1x weekly	17 (15.3)
1x weekly	62 (55.9)
2x weekly	29 (26.1)
Other	3 (2.7)
Unspecified or Intermittent	0 (0.0)

FVIII, factor VIII; FIX, factor IX; rFIX, recombinant factor IX product.

Supplemental Table S10. Incidence and severity of adverse events: hemophilia B cohort (*n* = 111)—safety analysis set.

	***n* (%)**			
**MedDRA Preferred Term**	**Mild**	**Moderate**	**Severe**	**Total**
Any AE	8 (7.2)	9 (8.1)	5 (4.5)	22 (19.8)
Appendicitis	0	0	1 (0.9)	1 (0.9)
Arthralgia	2 (1.8)	0	0	2 (1.8)
Arthropathy	0	1 (0.9)	0	1 (0.9)
Back pain	1 (0.9)	0	0	1 (0.9)
Complex regional pain syndrome	1 (0.9)	0	0	1 (0.9)
Contusion	0	1 (0.9)	0	1 (0.9)
COVID-19	1 (0.9)	0	0	1 (0.9)
Dermatitis allergic	0	1 (0.9)	0	1 (0.9)
Duodenal polyp	1 (0.9)	0	0	1 (0.9)
Enlarged uvula	0	1 (0.9)	0	1 (0.9)
Enteritis	0	1 (0.9)	0	1 (0.9)
Erysipelas	1 (0.9)	0	0	1 (0.9)
Fall	0	1 (0.9)	0	1 (0.9)
Gastrointestinal hemorrhage	0	2 (1.8)	0	2 (1.8)
Hemophilic arthropathy	0	0	1 (0.9)	1 (0.9)
Hematoma	0	2 (1.8)	0	2 (1.8)
Hematospermia	0	1 (0.9)	0	1 (0.9)
Hematuria	1 (0.9)	0	0	1 (0.9)
Hepatitis acute	0	1 (0.9)	0	1 (0.9)
Joint injury	0	1 (0.9)	0	1 (0.9)
Ligament rupture	0	0	1 (0.9)	1 (0.9)
Ligament sprain	1 (0.9)	0	1 (0.9)	2 (1.8)
Microcytic anemia	0	0	1 (0.9)	1 (0.9)
Miliaria	1 (0.9)	0	0	1 (0.9)
Muscle hemorrhage	0	1 (0.9)	0	1 (0.9)
Nasal congestion	1 (0.9)	0	0	1 (0.9)
Nasopharyngitis	1 (0.9)	0	0	1 (0.9)
Nephrolithiasis	1 (0.9)	0	0	1 (0.9)
Paresthesia	0	1 (0.9)	0	1 (0.9)
Plantar fasciitis	0	1 (0.9)	0	1 (0.9)
Pneumonitis	0	0	1 (0.9)	1 (0.9)
Post procedural infection	0	1 (0.9)	0	1 (0.9)
Rhinorrhea	1 (0.9)	0	0	1 (0.9)
Road traffic accident	1 (0.9)	0	0	1 (0.9)
Sinus tachycardia	1 (0.9)	0	0	1 (0.9)
Tendon disorder	1 (0.9)	0	0	1 (0.9)
Tendonitis	1 (0.9)	0	0	1 (0.9)
Tooth fracture	0	1 (0.9)	0	1 (0.9)
Upper respiratory tract infection	1 (0.9)	0	0	1 (0.9)
Wrist fracture	0	1 (0.9)	0	1 (0.9)

Note: If a participant had more than one occurrence in the same preferred term event category, only the most severe occurrence was counted. Participants were counted only once per System Organ Class and Preferred Term. Includes all data collected from the time the participant provides informed consent through the duration of the study until their final study or early termination visit. MedDRA v27.1 coding dictionary was applied.

Abbreviations: AE, adverse event MedDRA, Medical Dictionary for Regulatory Activities.

Supplemental Table S11. Incidence and severity of serious adverse events: hemophilia B cohort (*n* = 111)—safety analysis set.

	***n* (%)**			
**MedDRA Preferred Term**	**Mild**	**Moderate**	**Severe**	**Total**
Any SAE	0	5 (4.5)	4 (3.6)	9 (8.1)
Appendicitis	0	0	1 (0.9)	1 (0.9)
Arthropathy	0	1 (0.9)	0	1 (0.9)
Enteritis	0	1 (0.9)	0	1 (0.9)
Gastrointestinal hemorrhage	0	2 (1.8)	0	2 (1.8)
Hemophilic arthropathy	0	0	1 (0.9)	1 (0.9)
Joint injury	0	1 (0.9)	0	1 (0.9)
Ligament rupture	0	0	1 (0.9)	1 (0.9)
Ligament sprain	0	0	1 (0.9)	1 (0.9)
Pneumonitis	0	0	1 (0.9)	1 (0.9)
Tooth fracture	0	1 (0.9)	0	1 (0.9)

Note: If a participant had more than one occurrence in the same preferred term event category, only the most severe occurrence was counted. Participants were counted only once per System Organ Class and Preferred Term. Includes all data collected from the time the participant provides informed consent through the duration of the study until their final study or early termination visit. MedDRA v27.1 coding dictionary was applied.

MedDRA, Medical Dictionary for Regulatory Activities; SAE, serious adverse event.
